# DR6 as a Diagnostic and Predictive Biomarker in Adult Sarcoma

**DOI:** 10.1371/journal.pone.0036525

**Published:** 2012-05-02

**Authors:** Kun Yang, Colin Mooney, Greg Spahlinger, Scott Schuetze, Hugo Arias-Pulido, Claire Verschraegen, Phyllis Gimotty, Ronald J. Buckanovich

**Affiliations:** 1 Division of Hematology Oncology, Department of Medicine, University of Michigan, Ann Arbor, Michigan, United States of America; 2 Division of Hematology-Oncology, Department of Internal Medicine, University of New Mexico, Albuquerque, New Mexico, United States of America; 3 Department of Biostatistics, University of Pennsylvania, Philadelphia, Pennsylvania, United States of America; 4 Division of Gynecologic Oncology, Department of Obstetrics and Gynecology, University of Michigan, Ann Arbor, Michigan, United States of America; Sun Yat-sen University Medical School, China

## Abstract

**Background:**

The Death Receptor 6 (DR6) protein is elevated in the serum of ovarian cancer patients. We tested DR6 serum protein levels as a diagnostic/predictive biomarker in several epithelial tumors and sarcomas.

**Methods:**

DR6 gene expression profiles were screened in publically available arrays of solid tumors. A quantitative immunofluorescent western blot analysis was developed to test the serum of healthy controls and patients with sarcoma, uterine carcinosarcoma, bladder, liver, and pancreatic carcinomas. Change in DR6 serum levels was used to assay the ability of DR6 to predict the response to therapy of sarcoma patients.

**Results:**

DR6 mRNA is highly expressed in all tumor types assayed. Western blot analysis of serum DR6 protein demonstrated high reproducibility (r = 0.97). Compared to healthy donor controls, DR6 serum levels were not elevated in patients with uterine carcinosarcoma, bladder, liver, or pancreatic cancers. Serum DR6 protein levels from adult sarcoma patients were significantly elevated (p<0.001). This was most evident for patients with synovial sarcoma. Change in serum DR6 levels during therapy correlated with clinical benefit from therapy (sensitivity 75%, and positive predictive value 87%).

**Conclusion:**

DR6 may be a clinically useful diagnostic and predictive serum biomarker for some adult sarcoma subtypes.

**Impact:**

Diagnosis of sarcoma can be difficult and can lead to improper management of these cancers. DR6 serum protein may be a tool to aid in the diagnosis of some sarcomatous tumors to improve treatment planning. For patients with advanced disease, rising DR6 levels predict non-response to therapy and may expedite therapeutic decision making and reduce reliance on radiologic imaging.

## Introduction

The death receptor (DR) proteins, a subset of the tumor necrosis factor (TNF) receptors super-family, have been implicated as serum biomarkers for solid tumors [1], [2]. TNF receptor proteins are present in tumor endothelial cells, tumor-associated myeloid cells, and tumor cells with variable levels of expression. A primary function for death receptors is to induce apoptosis [3]. Abnormal expression, regulation, or function of TNF receptors have been strongly implicated in autoimmune disease, osteoporosis, and cancer [4], [5], [6], [7], [8], [9], [10]. Six different death receptors are currently known.

The most recently identified TNF receptor is Tumor Necrosis Factor Receptor Superfamily Member 21 (TNFRSF21), also known as death receptor-6 (DR6). The function of DR6 in cancer is not entirely clear [11]. DR6 retains the characteristics of other family members, including a cysteine-rich extracellular domain and conserved intracellular death domain required for induction of cell death. Thus, like other death receptor proteins, DR6 has been implicated in the induction of apoptosis [12]. Additionally, DR6 may regulate the cytokine-driven differentiation of monocytes to dendritic cells, which suggests DR6 could play a role in the development of myeloid derived suppressor cells within tumors [11].

We recently identified DR6 as a potential serum tumor marker in ovarian cancer [1]. In addition to its expression in ovarian cancer, DR6 has been reported to be up-regulated in numerous solid tumors [13]. DR6 is expressed not only in cancer cells, but also in tumor vascular cells. This expression on host cells in the tumor microenvironment suggests DR6 may have broad applicability as a tumor biomarker. The role of DR6 as a biomarker in non-ovarian tumors has not heretofore been investigated.

We report here an analysis of DR6 as a potential biomarker in several non-ovarian tumors. In particular we analyzed the role of DR6 as a potential serum biomarker in adult sarcoma. Sarcomas in the adult are rare but relatively deadly. Unlike other malignancies, there are no clinically used serum biomarkers to suggest a potential mass may represent sarcoma. Similarly, there are no serum biomarkers which can be used with confidence to predict whether a patient receiving therapy is or is not gaining clinical benefit. Our studies suggest that serum DR6 levels are elevated in patients with some sarcomas. In addition, declining DR6 levels may identify those patients gaining clinical benefit from systemic therapy.

**Figure 1:**
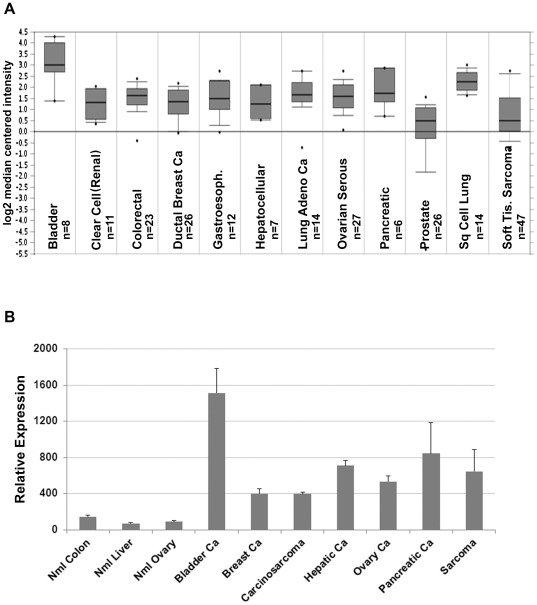
DR6 mRNA expression. Expression of DR6 mRNA in cancer expression arrays. A. DR6 mRNA expression in microarrays of the indicated tumor types determined using Oncomine™ software. Thick lines indicate the median, thin lines indicate the 90^th^/10^th^ percentiles, box indicates 25^th^–75^th^ percentiles, dots indicate the minimum and the maximum. B. Quantitative PCR confirming DR6 mRNA expression levels in the indicated tumor types. Average expression with standard error are indicated (n = 2–5 tumors in each group).

## Materials and Methods

### Gene Expression

We screened the gene expression profile of numerous tumor types using publically available array data [14], [15]. Oncomine™ (Compendia Bioscience, Ann Arbor, MI) was used for analysis and visualization. Data sets were analyzed independently and then combined with the normalized log 2 median centered intensity of zero.

Tumor tissues were obtained using IRB approved tumor banking protocols at the University of Michigan and the Cooperative Human Tissue Network. Tissues included normal colon (n = 3), normal liver (n = 2), normal ovary (n = 5), bladder cancer (n = 2), breast cancer (n = 5), carcinosarcoma (n = 3), hepatic cancer (n = 2), ovarian cancer (n = 5), pancreatic cancer (n = 3), and soft tissue sarcoma (n = 3 leiomyosarcoma, n = 2 uterine sarcoma). RNA was extracted from fresh frozen tissues using TRIzol per manufacturer's recommendations (Invitrogen, Grand Island NY) and qRT-PCR was then performed as previously described [1].

**Figure 2:**
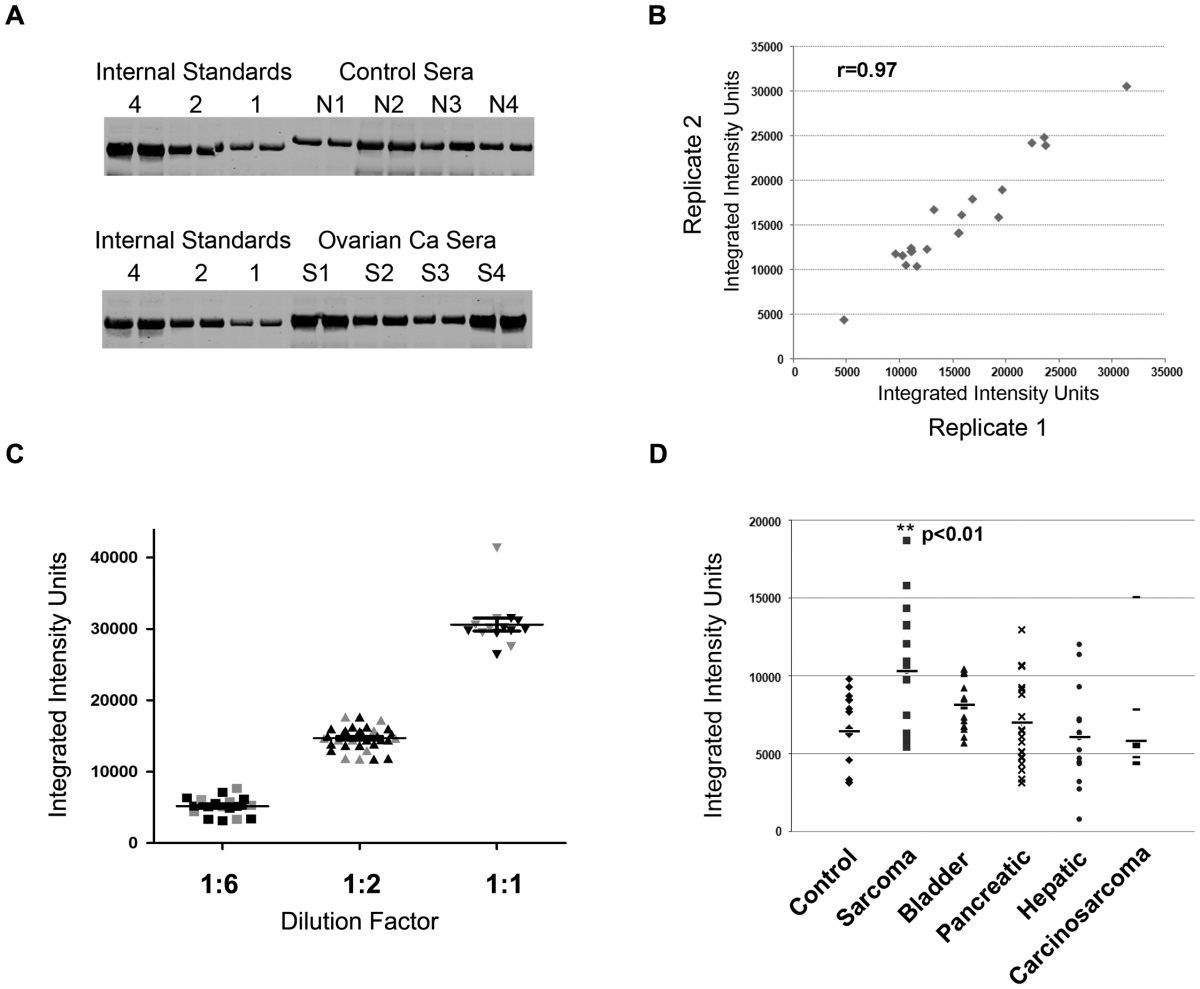
Development of a quantitative western blot assay for DR6. **A**. Representative quantitative immunofluorescent western blot analysis of DR6 serum protein expression from ovarian cancer patients and donor control patients. **B**. Concordance of western blot analysis of replicate samples in independent experiments (r = 0.97). **C**. Concordance of inter-day (gray) and intra-day (black) replicates of reference serum standards. **D**. Serum protein levels from control and patients with the indicated cancer types. Only adult sarcoma patients demonstrated a statistically significant increased expression of serum DR6 protein compared to control.

### Patients

Written informed consent was obtained from all patients prior to sample collection. Protocols for serum collection were reviewed and approved by the University of Michigan Institutional Review Board or the University of New Mexico Institutional Review Board (IRB). All clinical investigation was conducted according to the principles expressed in the Declaration of Helsinki. Five milliliters of blood was collected by venipuncture directly into serum separator tubes, centrifuged at 3000 RPM for 12 minutes, and then aliquoted into polypropylene vials and frozen at −70 degrees Celsius until use. Sera from patients with either carcinosarcomas or cancers of the bladder, liver, or pancreas were collected preoperatively as part of IRB approved University of Michigan serum banking protocols.

**Table 1:** Statistical analysis of fluorescent western blot assay replicates.

Serum Dilution	1∶6	1∶2	1∶1
Number of times assayed	18	36	14
			
Minimum	3130	11752	26411
25% Percentile	4165	13831	29478
Median	5183	14832	30044
75% Percentile	6119	15694	31214
Maximum	7688	17657	41317
			
Mean	5177	14701	30616
Standard Deviation	1299	1517	3394
Standard Error	306.3	252.8	907.1
Lower 95% Confidence Interval of mean	4531	14187	28656
Upper 95% Confidence Interval of mean	5823	15214	32575

Serum from patients with sarcoma was available from a total of 71 patients participating in IRB approved clinical trials. Serum was obtained from all 71 patients prior to trial initiation. 22 of these patients were participating in a therapeutic clinical trial at the University of New Mexico for chemotherapy-naïve patients. This serum was collected prior to initiation of either neo-adjuvant therapy or salvage therapy for metastatic disease. 49 of the patients were participating in a phase II clinical study of cyclophosphamide and sirolimus in patients with previously treated advanced adult sarcoma. A second sample was obtained from 41 patients at the University of Michigan after one cycle of therapy; two of these patients later donated a third sample after completing two cycles of therapy. Patients on trial were evaluated for progression via radiographic imaging every 2 months. Finally, control serum was collected as part of an IRB approved protocol, primarily from healthy volunteers age 18–65. Approximately 20% of control sera were collected preoperatively from patients who were ultimately diagnosed with benign ovarian conditions, including fibroadenoma, benign follicular cysts, cystadenomas, and endometriotic cysts.

**Figure 3:**
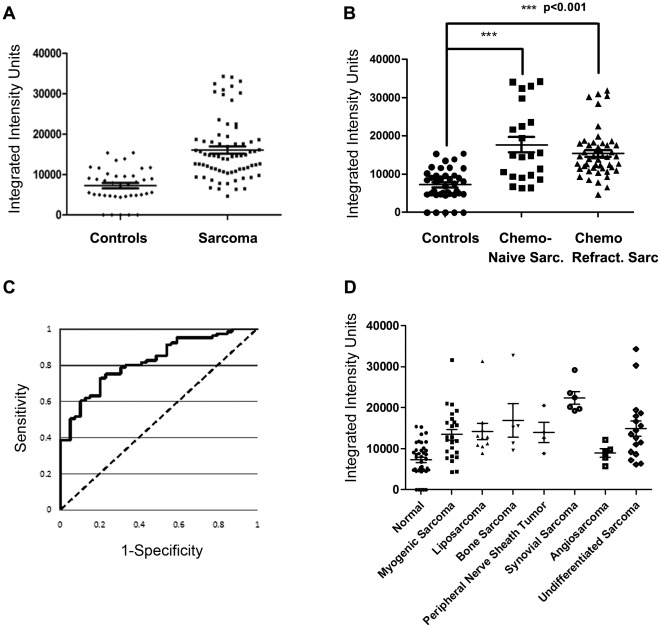
Sarcoma patients have significant elevation of DR6 serum protein. **A.** DR6 protein expression in a panel of 39 healthy controls and 71 patients with adult sarcoma. **B** DR6 levels in healthy controls and in sarcoma patients separated into chemo-naïve and chemo-refractory sub-groups. **C**. Receiver operator characteristic (ROC) curve for DR6 serum level in sarcoma patients versus controls. Area under the curve is 82.1%. **D**. Sarcoma patient DR6 serum protein levels based on histologic subtype. Averages are indicated by a long horizontal line. Brackets indicate standard deviations.

### Immunofluorescent western blot

A ‘master scale’ was developed with duplicate serial dilutions (0.25, 0.5, 1, 2, and 4 ul) of a single healthy control sera (GS) diluted into 40 µl PBS and then 15 µl of diluted serum was loaded with 5 µl of 2× loading buffer for PAGE. Immunofluorescent detection was performed using anti human DR6/TNFRSF21 affinity purified polyclonal goat IgG (1∶500, R&D Systems, Minneapolis, MN). Primary was detected with fluorescent donkey infrared dye 800CW conjugated anti-goat IgG (1∶5000 LiCor Biosciences, Lincoln NE). After washing, fluorescent images were captured with the Odyssey SA Infrared Imaging System (LiCor Biosciences) and quantified with LI-COR Odyssey Software version 2.1 per the manufacturer's instructions (http://biosupport.licor.com/docs/Odyssey_User_Guide_ver_3.0B.pdf). Captured digital images were quantified with using same size rectangle method to determine the integrated intensity value (pixel volume). Background values were subtracted from each experimental sample. Immunofluorescent western blot was run in duplicate for each and expression values indicated are averages of the two samples. Best linear fit was determined for the master scale using Microsoft XL. For each subsequent gel, experimental samples were run in a similar manner with 1 µl diluted into 40 ml of PBS, and a mini internal control sera scale (0.5, 1.0 and 2.0 µl) of GS sera was included for relative quantification. A normalized value was determined for each sample based in the internal control sample. The normalized value was then used in the linear correlation of the master gel to determine the relative expression value. Any samples with raw values outside the linear range of the assay (2000–25,000 expression units) were re-analyzed with additional dilutions. Dilution factor was taken into account after normalization.

**Table 2:** Characteristics of Sarcoma Patients Evaluated.

	Chemo- refractory	Chemo-Naive
**Median Age** – years (range)	57 (19–82)	54 (17–75)
**Male/Female**	28/21	13/9
**Median prior lines of chemotherapy**	3	NA
(range)	(1–6)	
**Stage II/III/IV**	NA	2/7/13
**Sarcoma sub-type**		
Leiomyosarcoma	16	6
Liposarcoma	9	2
Undifferentiated Pleiomorphic	5	0
Osteosarcoma	4	0
Synovial sarcoma	3	3
Peripheral nerve sheath tumor	3	1
Rhabdomyosarcoma	2	0
Extraskeletal myxoid chondrosarcoma	2	1
Angiosarcoma	1	3
Undifferentiated NOS/Other	4	6
**Neoadjuvant/Metastatic**	0/49	10/12

NA-not applicable.

### Statistical analysis

Descriptive statistics for serum concentrations of DR6 were calculated for each subject using GraphPad Prism5 software (GraphPad Software, Inc., La Jolla, Ca.). Unpaired t-tests were used with the minimum level of significance taken as p<0.05. Logistic regression analysis was used to estimate the association between the DR6 serum levels and case status (sarcoma patients versus controls). The receiver operating characteristic (ROC) curve was computed for DR6 to evaluate it as a diagnostic biomarker and the area under the curve (AUC) was computed. Proc Logistic was used for this analysis (SAS Software, version 9.2, Cary, NC.).

**Figure 4:**
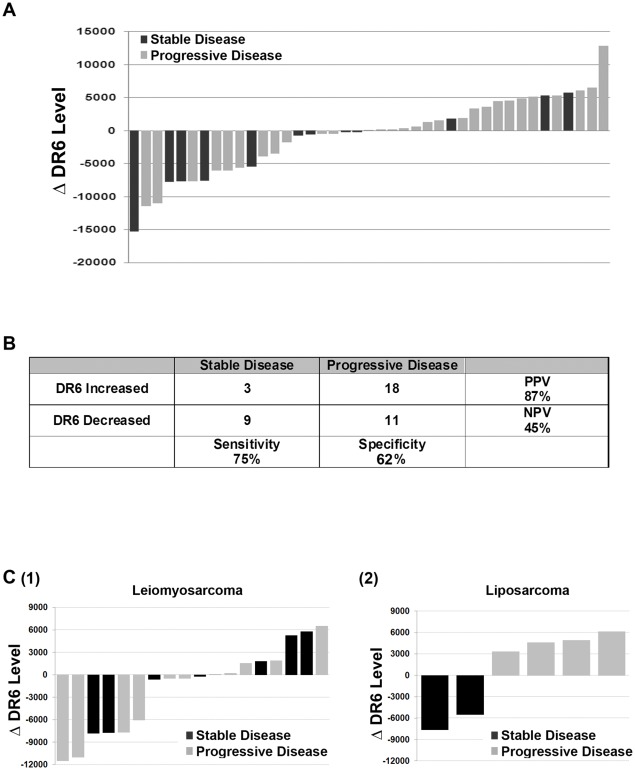
Correlating change in DR6 serum protein over time with response to therapy. **A**. Waterfall plot of change in DR6 serum protein values prior to and after therapy with sirolimus and cyclophosphamide in patients with advanced sarcoma; patients with stable disease are indicated by black bars and patients with progressive disease are indicated in Gray. **B**. Sensitivity, specificity, negative predictive value (NPV) and positive predictive value (PPV) for the change in DR6 serum protein levels to predict stable disease versus disease progression in sarcoma patients on therapy. **C**. Waterfall plot of change in DR6 serum protein values prior to and after therapy with sirolimus and cyclophosphamide in patients with (1) leiomyosarcoma and (2) liposarcoma. Patients with stable disease are indicated by black bars and patients with progressive disease are indicated in Gray.

## Results

### Death Receptor 6 gene expression and protein serum levels across various tumor types

In an attempt to identify tumors with high DR6 expression, we screened the gene expression profile of numerous tumor types using publicly available array data [14], [15]. DR6 expression was detectable in all tumor types, with the highest expression noted in bladder, pancreatic, and squamous cell lung cancers (Figure 1A). We next performed quantitative PCR for DR6 using cDNA from normal ovary, colon, and liver tissues and well as cDNA obtained from a panel of whole tumor samples. qRT-PCR confirmed increased DR6 mRNA levels in these tumors relative to the normal tissue controls, with the highest expression detected in bladder cancer, pancreatic cancer, hepatic cancer and adult sarcomas (Figure 1B).

We next developed a quantitative immunofluorescent western blot analysis approach to analyze DR6 protein levels. We used serum samples from ovarian cancer patients for which DR6 had previously been demonstrated to be elevated. Ovarian cancer serum samples were analyzed in duplicate together with internal standards to allow comparison between experiments. Analysis of duplicate samples within the same and different experiments revealed this method to be highly reproducible (R = 0.97, Figure 2A and B). Analysis of a minimum of 7 intra-day and 7 inter-day samples revealed a high degree of reproducibility (Figure 2C and Table 1). In addition, we found that up to 8 cycles of serum freezing/thawing had no impact on DR6 levels (data not shown).

Once the assay was confirmed, we screened DR6 serum protein levels from a panel healthy donor controls and a panel of patients with different tumor types including bladder cancer, hepatic cancer, pancreatic cancer, mullerian carcinosarcomas, and adult sarcomas. Interestingly, while qRT-PCR data demonstrated the highest DR6 mRNA levels in bladder cancer, pancreatic cancer, hepatic cancer and adult sarcomas, serum DR6 protein levels were statistically significantly elevated only in the serum of patients with adult sarcomas (Figure 2D). None of the other tumor types demonstrated a statistically significant increase in serum DR6 protein levels relative to healthy controls.

### DR6 serum protein level in patients with sarcoma versus control

Based on the preliminary screen above, serum DR6 levels were then compared using a panel from 71 adult sarcoma patients and 39 healthy controls. Table 2 summarizes the patient characteristics. 22 patients were chemotherapy naïve, with 10 of these patients receiving neo-adjuvant therapy for localized disease; the remaining 12 patients had metastatic disease. 49 patients had metastatic chemo-refractory disease, having received an average of 3 previous lines of chemotherapy. We observed a 2.2 fold increase in DR6 levels relative to normal controls (controls 0–15376 integrated intensity units, all sarcoma 4289–34298 integrated intensity units). More than 40% of sarcoma samples were found to be higher than the maximum expression in the normal control set (Figure 3A). Similar results were found for both chemo-naïve and chemo-refractory patients (Figure 3B). The association between case status (sarcoma patient versus control) and DR6 was statically significant (odds ratio = 1.3, 95% CI = 1.17 to 1.46, p<0.001). There was a 1.3-fold increase in the odds that a patient was a sarcoma patient for the group of patients with a 10,000 unit increase in DR6 compared to the odds for those at the base level. In addition, when DR6 was evaluated as a diagnostic marker with its ROC curve, the AUC was 82.1% (Figure 3C).

We further evaluated DR6 expression based on histologic subtypes, including myogenic sarcoma (22 leiomyosarcoma and 2 rhabdomyosarcoma), liposarcoma (n = 11), bone sarcoma (4 osteosarcomas and 1 Ewing sarcoma), peripheral nerve sheath tumor (n = 4), synovial sarcoma (n = 6), angiosarcoma (n = 5), and undifferentiated sarcoma (5 pleomorphic undifferentiated, 3 undifferentiated uterine, 6 undifferentiated-NOS, and 3 extra-skeletal myxoid chondrosarcomas) (Figure 3D). All groups, except angiosarcoma (p value = 0.386), retained a highly significant increase of serum DR6 compared to normal controls (p values<.0001–0.0044). The greatest difference among all subsets was seen in synovial sarcoma, which showed a 3.1 fold increase over normal controls. Bone sarcoma had the second greatest elevation (2.3 fold) followed by undifferentiated sarcoma (2.1 fold increase), liposarcoma (2.0 fold increase), and peripheral nerve sheath tumor and myogenic sarcoma, which both increased 1.9 fold.

### The role of DR6 in predicting disease progression

Clinical disease response information with pretreatment and on-treatment serum samples was available for 41 sarcoma patients. We next evaluated whether the change in DR6 levels before and after treatment correlated with disease response or clinical benefit. Thirty-nine patients had sera obtained pre-treatment and after 1 cycle of therapy, and 2 patients had an additional serum samples obtained after a second cycle. 12 of 41 patients demonstrated stable disease after 4 cycles (4 months) of therapy. Importantly, of the twelve patients with stable disease on therapy, 9 demonstrated a decrease in their DR6 levels (sensitivity = 75%, Figure 4A). 18 of 21 patients with increasing Dr6 levels demonstrated progressive disease within 2 months of starting treatment (positive predictive value = 85.7%). The negative predictive value and specificity were 45% and 62.1% respectively (Figure 4B). Similar results were obtained if samples which demonstrated <10% change in DR6 levels were considered as ‘stable disease’.

The only histological subtypes for which we had more than n = 4 samples in which to evaluate change in DR6 level in response to therapy were leiomyosarcoma (n = 18) and liposarcoma (n = 6). For leiomyosarcoma, change in DR6 serum protein after the first cycle of therapy did not appear to be a useful predictive biomarker (Fig. 4C(1)). For liposarcoma change in DR6 serum protein level after one cycle of therapy demonstrated 100% sensitivity and specificity to predict stable disease versus progressive disease (Fig. 4C(2)). Caution must be taken in interpreting this data given the small sample size.

## Discussion

Adult sarcoma is a rare but relatively deadly cancer [16]. The World Health Organization (WHO) describes more than 50 histological subtypes of soft tissue sarcomas associated with unique clinical features and biologic behavior [17]. Because of their rarity and diversity of presentation, sarcomas can be difficult to diagnose. This is so mainly because benign soft tissue masses outnumber sarcomas by a factor of at least 100 [17]. In the present study, we demonstrated that compared to healthy controls, adult patients with sarcoma have increased serum DR6 protein levels (p<0.001). We observed this in two independently collected sera banks. As a biomarker of sarcoma, DR6 could help to differentiate sarcomas from benign soft tissue masses. This would aid in appropriate surgical treatment planning, which is imperative because appropriate surgical management is associated with improved outcome [18]–[19]. Additional confirmatory studies are necessary.

Given the heterogeneity of sarcomas it seems unlikely that any tumor marker would be equally informative in all histologic subtypes. As a diagnostic tool, DR6 appears to have greatest potential in patients with synovial sarcoma; the lowest DR6 serum level in patients with synovial sarcoma measured well above that of all controls. Similarly, change in DR6 serum protein levels may serve as a predictive biomarker for some patients receiving chemotherapy. This potential appears greatest for patients with liposarcoma. However, given the small sample size in our study, prospective studies will be necessary to confirm this observation.

Our analysis of DR6 as a predictive biomarker is limited by the fact that only two time points were collected during therapy; at pre-treatment and before the second cycle of therapy. Biomarker ‘surge’, the initial spike of a marker measured following a cycle of effective treatment, could confound results when evaluating values of only two early, short-interval time points. Analysis of DR6 trend from pretreatment to later cycles of therapy could be more informative [20]. In fact, in our study two patients did provide serum for analysis prior to the third cycle of systemic therapy. One patient had progressive disease with a slight decrease in DR6 at the second visit; however, there was a dramatic increase in DR6 with the third serum sample. Another patient had stable disease with four cycles of therapy and showed an increase in DR6 after the first cycle of therapy, but then showed a precipitous decline after the second cycle. Further studies with serial evaluation of DR6 as a biomarker of response in the setting of standard sarcoma chemotherapy are necessary. Finally, if this is to be developed as a clinical assay, an ELISA assay that can easily be used for bulk testing would be beneficial. Unfortunately, we have found the currently available antibodies unsuitable for ELISA. While many of these antibodies work with denatured DR6 on western blot, or with ELISA for bacterially produced DR6 fusion proteins, we found that the currently available antibodies were not successful in recognizing native DR6 in patient serum. Ultimately, new antibodies will be necessary to recognize native DR6.

Surprisingly, DR6 protein in sera did not correlate with mRNA expression levels in tumors (data not shown). Thus DR6 serum protein level may not be regulated at the level of mRNA. Consistent with this, it was recently shown that soluble DR6 is the result of matrix metalloproteinase 14 (MMP-14) cleavage of membrane bound DR6 [21]. Thus serum DR6 protein may serve as an indicator of tumor MMP-14 levels. MMP-14 has been correlated with tumor invasion, metastases, and poor patient prognosis [22].

### Summary

DR6 serum protein levels demonstrate a 2–3 fold increase in patients with sarcoma, relative to normal healthy controls. A retrospective analysis of DR6 serum protein levels suggests that DR6 may be a biomarker for disease progression in patients with sarcoma. These studies support prospective evaluation of DR6 as a predictive biomarker in sarcoma patients undergoing treatment with standard chemotherapy.
